# Natural Biflavonoids Modulate Macrophage–Oxidized LDL Interaction *In Vitro* and Promote Atheroprotection *In Vivo*

**DOI:** 10.3389/fimmu.2017.00923

**Published:** 2017-08-04

**Authors:** Jorge H. Tabares-Guevara, Oscar J. Lara-Guzmán, Julian A. Londoño-Londoño, Jelver A. Sierra, Yudy M. León-Varela, Rafael M. Álvarez-Quintero, Edison J. Osorio, José R. Ramirez-Pineda

**Affiliations:** ^1^Grupo Inmunomodulación, Facultad de Medicina, Universidad de Antioquia, Medellín, Colombia; ^2^Grupo de Investigación en Sustancias Bioactivas, Facultad de Ciencias Farmacéuticas y Alimentarias, Universidad de Antioquia, Medellín, Colombia

**Keywords:** atherosclerosis, oxidized LDL, macrophage, foam cell, biflavonoid, morelloflavone, volkensiflavone, *Garcinia madruno*

## Abstract

The accumulation of oxidized ApoB-100-containing lipoproteins in the vascular intima and its subsequent recognition by macrophages results in foam cell formation and inflammation, key events during atherosclerosis development. Agents targeting this process are considered potentially atheroprotective. Since natural biflavonoids exert antioxidant and anti-inflammatory effects, we evaluated the atheroprotective effect of biflavonoids obtained from the tropical fruit tree *Garcinia madruno*. To this end, the pure biflavonoid aglycones morelloflavone (Mo) and volkensiflavone (Vo), as well as the morelloflavone’s glycoside fukugiside (Fu) were tested *in vitro* in primary macrophages, whereas a biflavonoid fraction with defined composition (85% Mo, 10% Vo, and 5% Amentoflavone) was tested *in vitro* and *in vivo*. All biflavonoid preparations were potent reactive oxygen species (ROS) scavengers in the oxygen radical absorbance capacity assay, and most importantly, protected low-density lipoprotein particle from both lipid and protein oxidation. In biflavonoid-treated macrophages, the surface expression of the oxidized LDL (oxLDL) receptor CD36 was significantly lower than in vehicle-treated macrophages. Uptake of fluorescently labeled oxLDL and cholesterol accumulation were also attenuated in biflavonoid-treated macrophages and followed a pattern that paralleled that of CD36 surface expression. Fu and Vo inhibited oxLDL-induced ROS production and interleukin (IL)-6 secretion, respectively, whereas all aglycones, but not the glucoside Fu, inhibited the secretion of one or more of the cytokines IL-1β, IL-12p70, and monocyte chemotactic protein-1 (MCP-1) in lipopolysaccharide (LPS)-stimulated macrophages. Interestingly, in macrophages primed with low-dose LPS and stimulated with cholesterol crystals, IL-1β secretion was significantly and comparably inhibited by all biflavonoid preparations. Intraperitoneal administration of the defined biflavonoid fraction into ApoE^−/−^ mice was atheroprotective, as evidenced by the reduction of the atheromatous lesion size and the density of T cells and macrophages infiltrating the aortic root; moreover, this treatment also lowered the circulating levels of cholesterol and the lipid peroxidation product malondialdehyde. These results reveal the potent atheroprotective effects exerted by biflavonoids on key events of the oxLDL–macrophage interphase: (i) atheroligand formation, (ii) atheroreceptor expression, (iii) foam cell transformation, and (iv) prooxidant/proinflammatory macrophage response. Furthermore, our results also evidence the antioxidant, anti-inflammatory, hypolipemiant, and atheroprotective effects of *Garcinia madruno*’s biflavonoids *in vivo*.

## Introduction

Atherosclerotic cardiovascular diseases (CVD) are the current major human killers worldwide. Indexes of incidence, prevalence, mortality, and economic impact of CVD (1) make them priority target diseases for public health systems and the scientific community. Key events in the atherosclerotic pathophysiology are (1) dyslipidemia-associated lipoprotein retention in the subintimal space of particular anatomical regions of the arterial tree, (2) physicochemical modifications of lipoproteins leading to endothelial dysfunction and activation, recruitment of leukocytes, and the subsequent triggering of inflammatory cascades governed by innate and adaptive immune systems, and (3) uncontrolled vicious circle of inflammation and lipoprotein accumulation/oxidation leading to lesion growth, inestabilization, and eventually to clinical events (2). Both non-hematopoietic and hematopoietic vascular cells participate in atherogenesis. On the one hand, estructural cells of the arterial wall, such as endothelial cells (ECs) and smooth muscle cells (SMCs), play important roles in various events of the atherogenic response, such as mechanotransduction and vessel remodeling, respectively (3). On the other hand, the macrophage, a bone marrow-derived cell, appears to play a central role in all stages of the atherosclerotic process. For instance, the recognition of modified low-density lipoprotein (LDL) by macrophages *via* innate receptors, such as CD36, and the subsequent triggering of signaling pathways leading to foam cell formation, inflammation, proliferation, or cell death are considered essential proatherogenic events (3). Recent research emphasizes the importance of this oxidized LDL (oxLDL)-macrophage interphase for atherosclerosis (4) and validates the associated events as promising therapeutic targets (5–8).

Flavonoids are a widely distributed class of phytochemicals present in many vegetables and fruits, with undisputed health-promoting properties. Although clinical, epidemiological, and experimental data have revealed the potential of this compounds for prevention or treatment of many diseases, including CVD (9–11), the underlying putative mechanisms are multiple, complex, and controversial (11, 12). In spite of this, there is an evident expectation that a better understanding of the mode of action of these intriguing molecules will guide their future intelligent exploitation from a dietary and/or pharmacological perspective (13). Biflavonoids are a class of flavonoids, which consist of flavonoid dimers formed by the covalent bond (C-C or C-O-C) between two monoflavonoids. Biflavonoids are also secondary metabolites but with a more restricted presence in plants, serving as chemotaxonomic markers for several species (14, 15). An array of biological activities, largely overlapping with those of flavonoids, has been reported for biflavonoids and biflavonoid-enriched preparations from plants (15–17). Among them, antioxidant, antiproliferative, or anti-inflammatory activities appear prominent, suggesting their potential for pharmacological application in the prevention or treatment of atherosclerosis and associated vascular diseases. However, surprisingly, very little has been investigated regarding the atheroprotective effects of biflavonoids. A remarkable exception is the study by Pinkaew and collaborators, in which atherosclerosis-prone Ldlr^−/−^ Apobec1^−/−^ mice were fed morelloflavone (Mo, a prototypic biflavonoid)-supplemented diet and a significant reduction in the size of atherosclerotic lesions was observed (18). Two studies reported that Mo altered vascular SMC and EC migration *in vitro* and inhibited neointimal formation and tumor angiogenesis *in vivo* (19, 20), suggesting that some atheroprotective effects of this biflavonoid could be explained by actions on non-hematopoietic vascular cells. Although some studies have evidenced the effects of biflavonoids on the inflammatory response of mouse and human macrophages (16), to our knowledge, there are no reports on the effects of Mo or other biflavonoid in the context of the oxLDL–macrophage interaction.

Genus *Garcinia* has been reported to be the main source of biflavonoids and these compounds are recognized as chemotaxonomic markers of *Garcinia* species (14). A previous phytochemical study with endemic species from northern South America revealed the abundance of rotameric biflavonoids in the aerial parts of the tropical fruit tree *Garcinia madruno* (21). Several biflavonoids, such as Mo, volkensiflavone (Vo), or Amentoflavone (Am), as well as some biflavonoid glycosides such as Fukugiside (Fu) were identified (21, 22). Considering the paucity of information regarding the atheroprotective effects and mechanisms of Mo in particular and of biflavonoids in general, as well as the importance of macrophages in atherogenesis, we investigated the effects of the most abundant biflavonoids isolated from *G. madruno* on the proatherogenic response of primary mouse macrophages. We found that Mo, Vo, and/or Fu were active *in vitro* at preventing LDL oxidation, modulating CD36 scavenger receptor (SR) expression, attenuating foam cell formation, and inhibiting inflammatory response in proatherogenic macrophages. Furthermore, in *in vivo* experiments using ApoE-deficient (ApoE^−/−^) mice, we found that intraperitoneal (i.p.) treatment with a defined biflavonoid fraction (BF) from *G. madruno* attenuated atherosclerosis development and this effect was associated with hypolipidemic and antioxidant activities as well as with reduced inflammatory macrophage and T cell infiltrate in the aortic root. Our results confirm the metabolic, antioxidant, and immunomodulatory effects of biflavonoids *in vitro* and *in vivo* and contribute to the understanding of their pharmacological activities.

## Materials and Methods

### Animals, Diet, and Reagents

**Figure 1:**
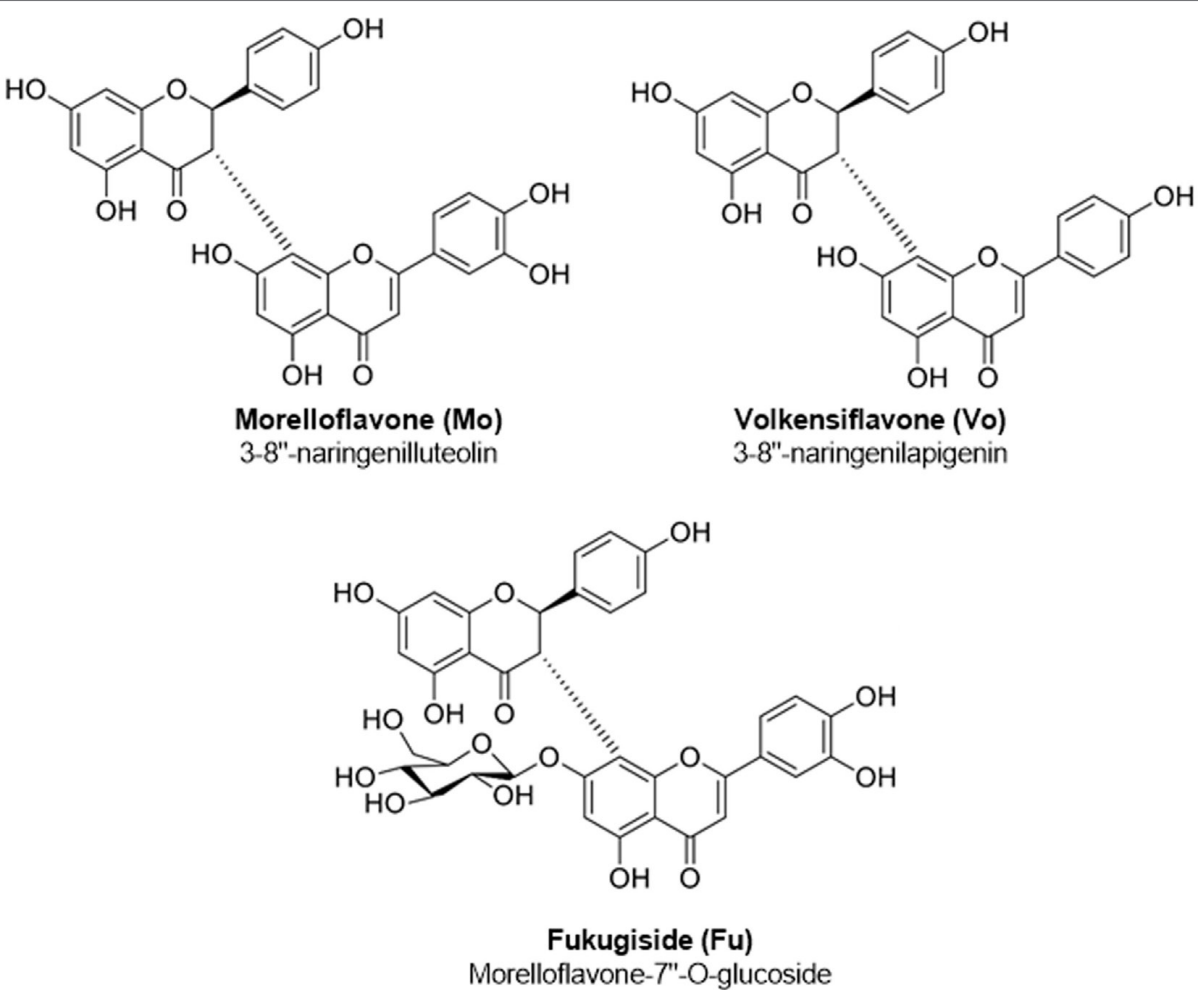
Chemical structures of biflavonoids used in the study.

Wild type and ApoE^−/−^ (B6.129P2-Apoe*^tm1Unc^*/J) C57BL/6 mice were obtained from Charles River (USA) and Jackson laboratories (USA), respectively. Mice were bred and maintained under specific pathogen-free (SPF) conditions at the SPF facility of the Sede de Investigacion Universitaria (SIU), Universidad de Antioquia. This study was carried out according to the guidelines for the care and use of laboratory animals; all of the procedures were approved by the institutional animal care committee (Comite institucional para el cuidado y uso de los animales, Universidad de Antioquia). Animals were fed a standard mouse diet (LabDiet, USA) or a high-fat diet (HFD, adjusted calories diet 42%, Harlan Teklad, USA). Lipopolysaccharide (LPS) (from *E. coli* 0727:B8), 2,7-diclorofluorescein diacetate (DCFH-DA), Fluorescein, 2,2′-Azobis (2-methylpropionamidine) dihydrochloride (AAPH), (±)-6-Hydroxy-2,5,7,8-tetramethylchromane-2-carboxylic acid (Trolox), 2-thiobarbituric acid (TBA), and 1,1,3,3-tetramethoxypropane were obtained from Sigma-Chemical Co. (USA). Cholesterol crystals were prepared following our previously published protocol (23). Human LDL was prepared and oxidized following published protocols (23, 24). For some experiments, oxLDL preparations (1 mg/mL) were fluorescently labeled with 1,1-dioctadecyl-3,3,3,3-tetramethylindocarbocyanine perchlorate (DiI; Invitrogen, USA) during 12 h and dialyzed against phosphate-buffered saline (PBS) for 12 h, as described previously (23). The aglycone Biflavonoids Mo and Vo, as well as the morelloflavone’s glucoside Fu (Figure 1) were prepared from the aerial parts of *G. madruno* and purified using batch chromatography as previously reported (21, 22). A portion of the ethyl acetate extract (50 g) from *G. madruno* was fractionated by size exclusion chromatography on Sephadex LH-20 and vacuum liquid column chromatography (CC). In this way, a considerable purification of a biflavonoid-rich fraction was achieved (2.2 g). The methanolic extract was subjected to silica gel CC to yield Fu. Extensive CC of BF produced Mo and Vo. The compounds were identified on the basis of 1D, 2D nuclear magnetic resonance (HMQC and HMBC) spectroscopic methods and chemical evidence (21, 25). For *in vivo* experiments, a BF of a known composition (Mo, Vo, and Am; with an approximate proportion of 85, 10, and 5% by weight, respectively; purity 94%, as determined by high-performance liquid chromatography) was also prepared according to the previously described method (21, 22). Stock solutions of pure Biflavonoids and the BF were prepared in dimethyl sulfoxide (DMSO) (1 mg/mL) and stored at −20°C for further use.

### Oxygen Radical Absorbance Capacity (ORAC) and LDL Oxidation Inhibition Assays

For the ORAC assay, AAPH was used as a peroxyl radical generator, Trolox as antioxidant standard, and fluorescein as the fluorescent probe (26). Fluorescein, AAPH, and the samples were prepared in a 75 mM phosphate buffer (pH 7.4). Pure biflavonoids, BF or Trolox standards (25 µL) were mixed with 150 µL of 1 µM fluorescein and preincubated at 37°C for 30 min before addition of 25 µL of AAPH solution (200 mM). The fluorescence (485 nm excitation/520 nm emission) was measured every 2 min for 120 min in a Spectra Max Gemini EM fluorimeter-Molecular devices (New Orleans, LA, USA). The relative ORAC values were calculated using the differences of areas under the decay curves and were expressed as µmol Trolox equivalents (TE) per μmol biflavonoid (μmol TE μmol^−1^ biflavonoid) or per gram BF (μmol TE g^−1^ BF). Based on previous work, a concentration of 10 μM was chosen to test pure biflavonoids (Mo, Vo, and Fu) in the ORAC assay. The BF was tested at 5.5 µg/mL (which equals 10 µM Mo, the dominant biflavonoid in the BF). The flavonoid quercetin (Qu; 10 µM) was used as a positive control of known antioxidant activity in some ORAC assays. For interpretation, the higher ORAC value of a compound/preparation, the higher antioxidant activity. To assess the ability of Biflavonoids to protect LDL from oxidation, methods that measure lipid peroxidation (thiobarbituric acid reactive substances, TBARS assay), and the ApoB-100 protein electronegativity (relative electrophoretic mobility, REM assay) in the LDL particle were used. For TBARS, LDL was incubated in potassium phosphate buffer (0.1 M) to a final protein concentration of 5 g/L and preincubated during 15 min with biflavonoid samples (10 µM biflavonoids or 5.5 µg/mL BF); finally, peroxidation reaction was initiated with CuSO_4_ (40 µM), incubated for 9 h at 37°C and stopped with 1% ethylenediaminetetraacetic acid. A TCA–TBA–HCl stock solution (15% w/v trichloroacetic acid; 0.67% w/v thiobarbituric acid; 0.1 N HCl) was added to the reaction mixture that was then heated at 95°C for 20 min and soon after centrifugated at 3,000 × *g* for 15 min and, finally, absorbances of supernatants were read at 532 nm. For malondialdehyde (MDA) calibration curve, different concentrations of 1,1,3,3-tetraethoxypropane were prepared as external standards. The oxidation of LDL was expressed as nanomole of MDA per mg LDL. For REM assays, LDL was preincubated as above using different concentrations of biflavonoids or BF and after 9 h of CuSO_4_-induced oxidation samples were taken to run in agarose gel electrophoresis (120 V, 500 mA) using barbital buffer (pH 8.7). Proteins were developed with Sudan black B staining (0.1% w/v in ethanol 95%).

### Macrophage Generation and Treatments

The *in vitro* effect of biflavonoids on macrophage proatherogenic responses [CD36 surface expression, foam cell formation, cholesterol accumulation, reactive oxygen species (ROS) production, and proinflammatory cytokine secretion] was assessed in primary bone marrow-derived macrophages, as previously reported (23). For this purpose, bone marrow cells were obtained from 8-week-old male C57BL/6 mice, cultured with granulocyte-macrophage colony-stimulating factor during 9 days and the resulting firmly adherent cells were removed with scrapers to be used as a source of macrophages for all the experiments. The culture medium was RPMI 1640 (Glutamax, Gibco, USA) containing 10% FBS, 100 U/mL penicillin, 100 µg/mL streptomycin and 0.05 β-mercaptoetanol and cells were incubated at 37°C with 5% CO_2_. In all cases, macrophages (5 × 10^5^ cells/mL) were pretreated with pure biflavonoids or the BF for 12 h and, subsequently, incubated with different stimuli (oxLDL, LPS, or cholesterol crystals). As negative controls, macrophages were treated with an equivalent volume of vehicle (DMSO 0.05% in PBS). None of the macrophage proatherogenic responses evaluated here were affected by incubations with vehicle in either basal or stimulated conditions (not shown). Likewise, biflavonoids alone did not induce any detectable effect on resting macrophages. All reagents used for macrophage differentiation and treatments were endotoxin free, as certified by the suppliers. Regular testing for endotoxin contamination was performed in macrophage cultures by using a colorimetric LAL assay (QCL-1.000, Lonza, USA) with negative results (less than 0.2 EU/mL). *In vitro* toxicity to the different treatments was assessed by a cell membrane integrity viability test (lactate dehydrogenase assay kit, LDH; Promega, USA).

### CD36 Expression, Foam Cell Formation, and Cholesterol Accumulation

Macrophage cultures preincubated with biflavonoids for 12 h (45 µM for Mo, Vo, and Fu, 25 µg/mL for BF) were further stimulated with oxLDL (30 µg/mL) during 36 h, washed and harvested with a cell scraper and cold PBS. Cell suspensions were then analyzed by flow cytometry to determine the surface density of the oxLDL receptor CD36. For the evaluation of foam cell formation, a cytometry-based fluorescent oxLDL uptake assay was used (23). Shortly, DiI-labeled oxLDL (50 µg/mL) was added to biflavonoid-pretreated macrophages and further incubated for additional 6 h. Cells were then removed, washed, and analyzed by flow cytometry. In parallel uptake assays, biflavonoids-pretreated cells were exposed to oxLDL (30 µg/mL) for additional 36 h, and the intracellular accumulation of cholesterol was determined by gas chromatography/mass spectrometry (GC/MS), using a standardized method (23) that quantifies total cellular cholesterol. Data are reported as total cholesterol per 10^6^ macrophages.

### ROS Production and Cytokine Secretion

Biflavonoid-pretreated macrophages (90 µM for Mo, Vo, and Fu, 50 µg/mL for BF) were washed twice with PBS and exposed to oxLDL (50 µg/mL) for 1 h. Cells were subsequently incubated with 10 µM fluorescent ROS-sensitive substrate DCFH-DA for 30 min, washed twice, resuspended with PBS, and finally analyzed by flow cytometry to assess intracellular ROS production. For cytokine secretion measurements, biflavonoid-pretreated macrophages (45 µM for Mo, Vo, and Fu, 25 µg/mL for BF) were stimulated with oxLDL (25 µg/mL) or LPS (10 µg/mL) for 12 additional hours. In another set of experiments, biflavonoid-pretreated macrophages were primed with LPS (10 ng/mL) for 2 h and exposed to cholesterol crystals (500 µg/mL) for additional 10 h, to complete a total time of treatment of 24 h. Macrophage culture supernatants were collected and used to quantify the levels of interleukin (IL)-1β, IL-6, IL-12p70, TNFα, MCP-1, and macrophage inflammatory protein (MIP)-1α by a multianalyte Luminex xMAP technology assay (Milliplex xMAP, Millipore, USA). The limit of detection was 3.2 pg/mL for all analytes.

### Flow Cytometry

For CD36 surface expression, cells were first incubated with a purified anti-mouse CD36 antibody (clone CRF D-2712, BD Biosciences, USA) or an isotype control (clone M18-254, BD Biosciences, USA) before incubation with a FITC-rat anti-mouse secondary antibody (clone C10-3, BD Biosciences, USA). For oxLDL uptake and ROS production, cells were resuspended in PBS and analyzed. A Beckman-Coulter EPICS XL flow cytometer was used for all acquisitions (at least 10,000 events), and data storage and analysis was performed with WinMDI software. The results were reported as the percentage of CD36^+^, DiI^+^, or DCF^+^ cells and the mean of fluorescence intensity (MFI).

### Assessment of the Atheroprotective Effect of Biflavonoids *In Vivo*

Six- to eight-week-old male ApoE^−/−^ mice (*n* = 12) were treated with i.p. injections of BF (70 mg/kg) every other day. After 2 weeks, mice were shifted to a HFD and maintained on BF treatments for additional 12 weeks. Given that the low oral bioavailability of biflavonoids (16) could lead to none/little systemic circulation of active compounds, i.p. administration was chosen for this study. A group of control mice were treated with a similar volume of vehicle (DMSO 0.1% in PBS). After 14 weeks, mice were sacrificed and the hearts/aortas were removed after *in situ* perfusion using PBS, and then fixed using 4% paraformaldehyde in PBS pH 7.4 for 48 h, immersed in 30% sucrose during 24 h and finally embedded with Shandon Cryomatrix™ (Thermo scientific, USA) to be stored at −20°C until use. Frozen hearts were processed with a cryostat (Leica Microsystems, Germany) to obtain 6–7-µm-thick sections of the aortic sinus as previously described (27). The sections were mounted on charged glass slides (Thermo Fisher Scientific, USA) and stained with conventional hematoxylin/eosin (H/E, Sigma-Aldrich, USA) or processed for immunohistochemistry (IHC). For IHC, aortic sinus sections were acetone-fixed, blocked for endogenous peroxidase activity, permeabilized, and incubated with a macrophage- or a T cell-specific mAb (anti-mouse monocyte/macrophage, clone MOMA-2, or anti-CD3 antibody, clone KT3, respectively; Serotec, UK). A secondary horseradish peroxidase-conjugated goat anti-rat IgG antibody and the chromogenic substrate 3,3-diaminobenzidine (Serotec, UK) were used to develop antibody binding. Finally, the sections were counterstained with hematoxylin. Microphotographs (40× magnification) were taken, and the area of the atherosclerotic lesion (H/E), macrophage infiltration (MOMA-2^+^), and T cell infiltration (CD3^+^) were calculated (in µm^2^) using the NIS Elements BR image analysis software (Nikon, Japan). Results were reported as the mean area of 9–12 sections per animal. Lipid deposition in aortas was determined by Oil Red O staining (OR, Sigma). Briefly, aortas were excised and fixed in 10%-buffered formalin. After adventitial tissue was carefully removed, aortas were opened longitudinally, stained with OR and pinned on a green wax surface. En face images were obtained under stereomicroscope (SMZ; Nikon, Japan) and analyzed by using Image J software. The luminal OR^+^ surface area was determined and expressed as pixels^2^ × 10^3^. Blood samples were also collected and serum levels of total cholesterol, hepatic enzymes aspartate transaminase (AST) and alanine transaminase (ALT), as well as amylase were determined using dry chemistry methods (Johnson and Johnson, USA) at the Universidad de Antioquia Veterinary School. Circulating levels of the lipid peroxidation product MDA were also determined in serum samples by the TBARS method and expressed in micromolars.

### Statistical Analysis

For *in vitro* assays, experiments were performed in triplicate and the mean ± SD was reported. One-way analyses of variance and Newman–Keuls *post hoc* tests were used for multiple group comparisons. For *in vivo* experiments, results were expressed as the mean ± SEM. Treated and control groups were compared using the unpaired Mann–Whitney or Student’s *t*-test. Results were analyzed using GraphPad Prism 5 software (GraphPad, USA). Statistically significant differences are indicated as follows: *p < 0.05, **p < 0.01, ***p < 0.001.

## Results

### Biflavonoids Attenuate the Formation of the Atherogenic Ligand oxLDL

**Figure 2:**
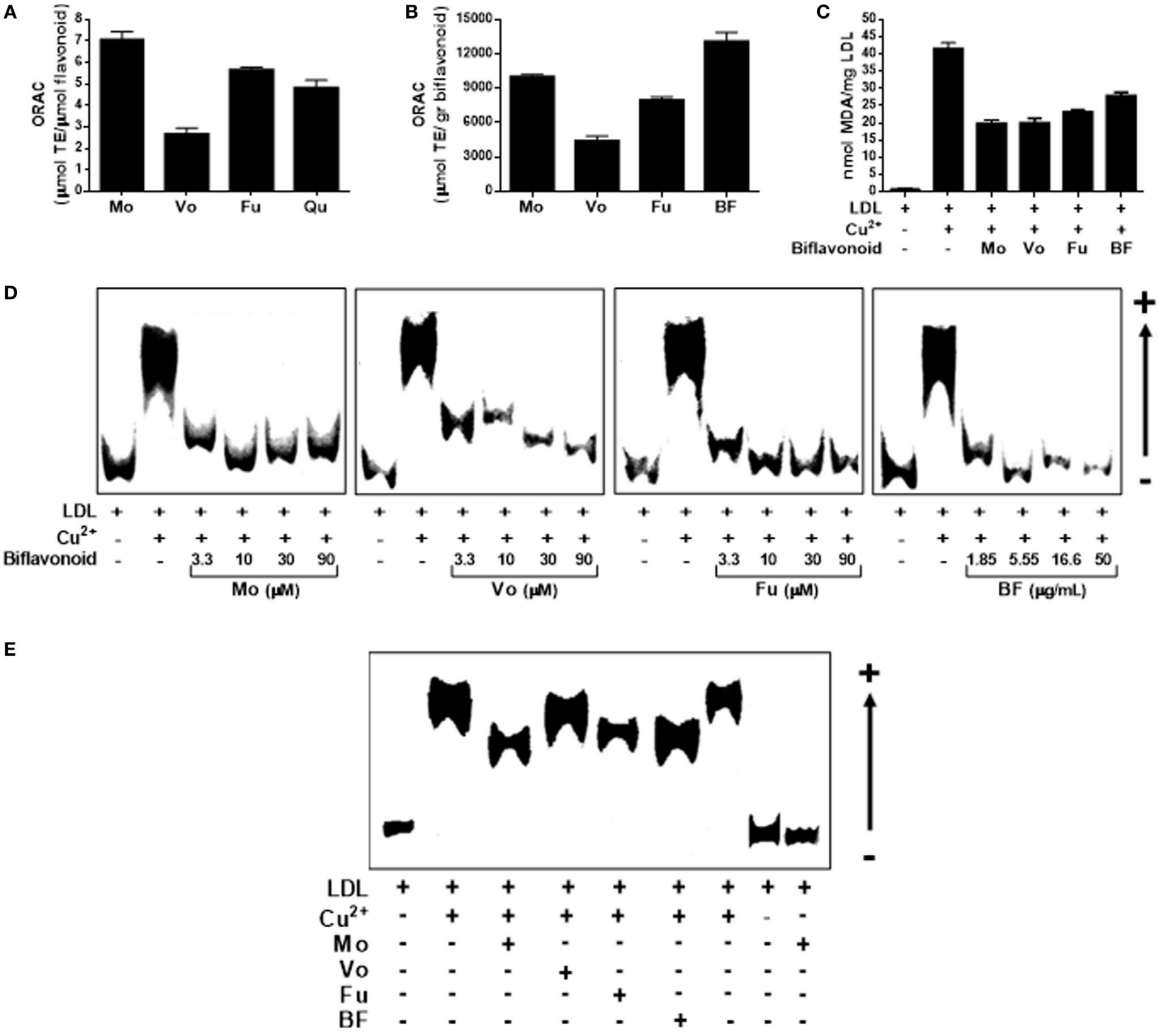
Biflavonoids inhibit low-density lipoprotein (LDL) oxidation. Oxygen radical absorbance capacity (ORAC) values of the biflavonoids Mo, Vo, and Fu (10 µM) were determined and expressed as micromole equivalents of Trolox per mol of biflavonoid to be compared with that of the monoflavonoid quercetin (Qu; 10 µM) **(A)**. ORAC value was also determined for the BF (5.5 µg/mL) and expressed as micromole equivalents of Trolox per gram to be compared with pure biflavonoids **(B)**. The effect of biflavonoids (10 µM for Mo, Vo, and Fu and 5.5 µg/mL for BF) on the Cu^2+^-induced formation of the lipid peroxidation product malondialdehyde in the LDL particle was assessed by TBARS assay **(C)**. Given the protective activity of biflavonoid preparations on LDL lipid peroxidation, a titration experiment of biflavonoids at the indicated concentrations (μM for Mo, Vo, and Fu; μg/mL for BF) on the electronegativity of the apolipoprotein fraction in the LDL was also determined by the REM assay in agarose gels **(D)**. Also, a comparison of the protective effect of low concentration (1.5 µM for Mo, Vo, and Fu, and 0.83 µg/mL for BF) biflavonoids on REM was performed with samples run in the same agarose gel **(E)**. Note that the biflavonoid Mo alone does not have prooxidant effect [last line in **(D)**] under the experimental conditions used. Results are representative of at least three independent experiments.

Low-density lipoprotein particle retention and subsequent oxidation in the subendothelial space is a crucial step in atherosclerosis initiation and progression. Therefore, prevention/attenuation of this step appears a logic mean to promote atheroprotection. We, therefore, began by investigating the antioxidant activity of biflavonoids in cell-free systems. First, we tested individual biflavonoids for their potential to inhibit peroxy-radical-induced oxidation by using the conventional ORAC method. As expected, all biflavonoids tested were potent antioxidants (Figure 2A). Mo and its glucoside Fu exhibited the highest TE (above quercetin, used as a reference polyphenol antioxidant). When ORAC results were transformed into μmol TE per gram, the highest antioxidant capacity was observed for the BF (Figure 2B), suggesting synergistic interactions among biflavonoids. We next investigated the antioxidant activity of biflavonoids in the more physiologically relevant system of Cu^2+^-induced LDL oxidation. When LDL oxidation was performed in the presence of any of the three pure biflavonoids, the formation of the lipid oxidation product MDA was reduced by approximately 50% (Figure 2C). The BF, although to a lesser extent, also protected LDL from Cu^2+^-induced oxidation (Figure 2C). Given the importance of the oxidation of the protein component in the LDL particle for the physiopathology of atherosclerosis, we also tested the potential of biflavonoids to prevent oxidation-induced increase of ApoB-100 electronegativity by REM assay. As shown in Figure 2D, all biflavonoids and the BF inhibited the accelerated migration of ApoB-100 in a dose-dependent manner. Treatment of LDL with low biflavonoid concentrations (1.5 µM) demonstrated a less pronounced effect for Vo (Figure 2E). Together, these results show that biflavonoids prevent LDL modification at lipid and protein levels and, consequently, reduce the formation of this key atherogenic ligand.

### Biflavonoids Inhibit the Expression of the Proatherogenic Receptor CD36 in Macrophages and Their Subsequent Transformation into Foam Cells

**Figure 3:**
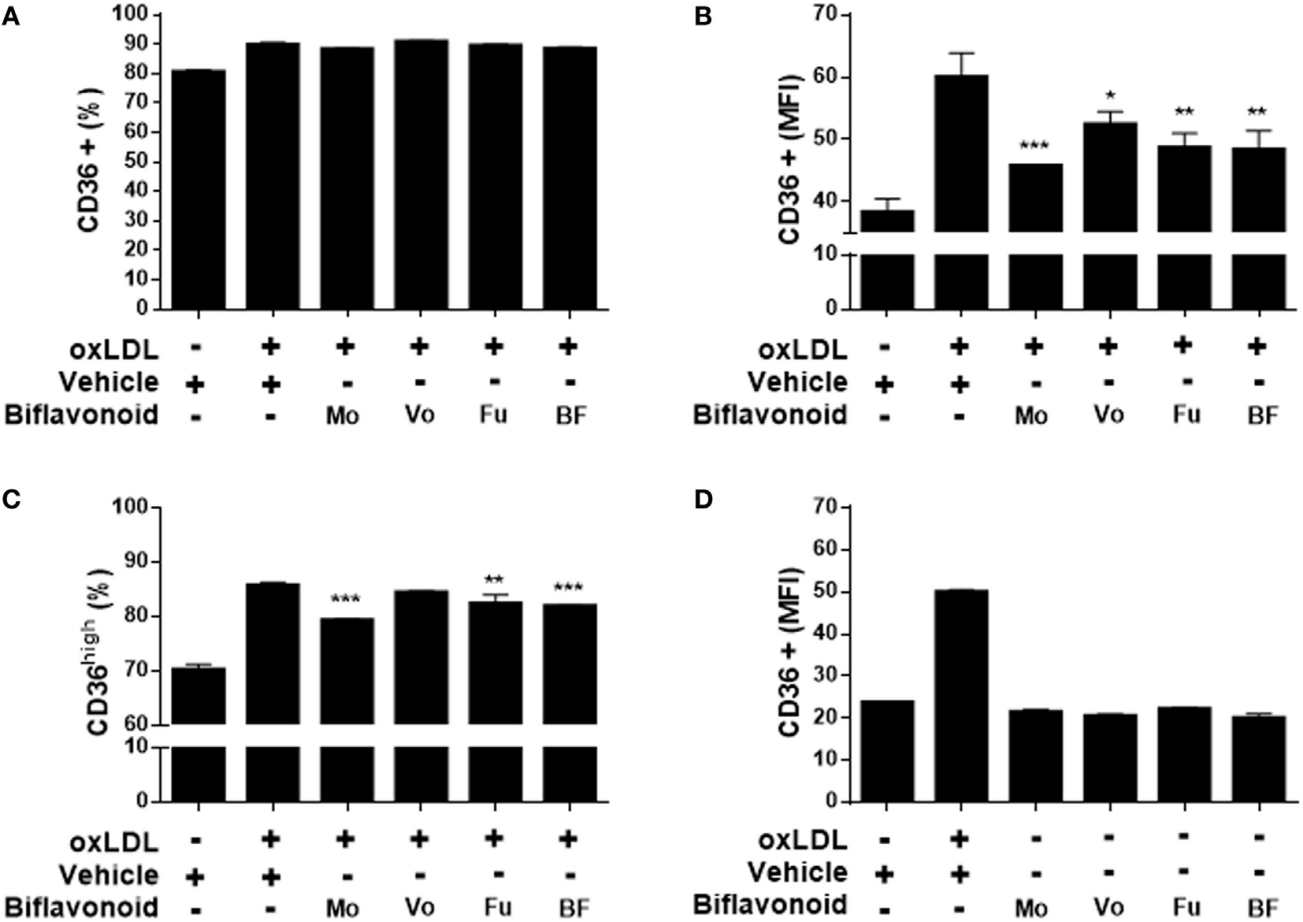
Biflavonoids inhibit surface CD36 expression on primary macrophages. Macrophages were preincubated 12 h with biflavonoids (45 µM for Mo, Vo, and Fu, and 25 µg/mL for BF) and, subsequently, incubated with oxLDL (30 µg/mL) for additional 36 h. The surface expression of CD36 was determined by flow cytometry and graphed as the percentage of CD36^+^ cells **(A)**, the MFI in CD36^+^ cells **(B)** or the percentage of CD36^high^ cells **(C)** based on a gating strategy shown in the Figure S2A in Supplementary Material. The effect of biflavonoid preparations on the expression of CD36 in resting macrophages was also determined and graphed as the MFI in CD36^+^ cells **(D)**. Experiments were performed in triplicate and bars represent the mean ± SD. *p < 0.05, **p < 0.01, ***p < 0.001, compared to vehicle-treated cells. The results are representative of at least three independent experiments. None of the treatments altered cell viability as determined by LDH release assay (not shown).

**Figure 4:**
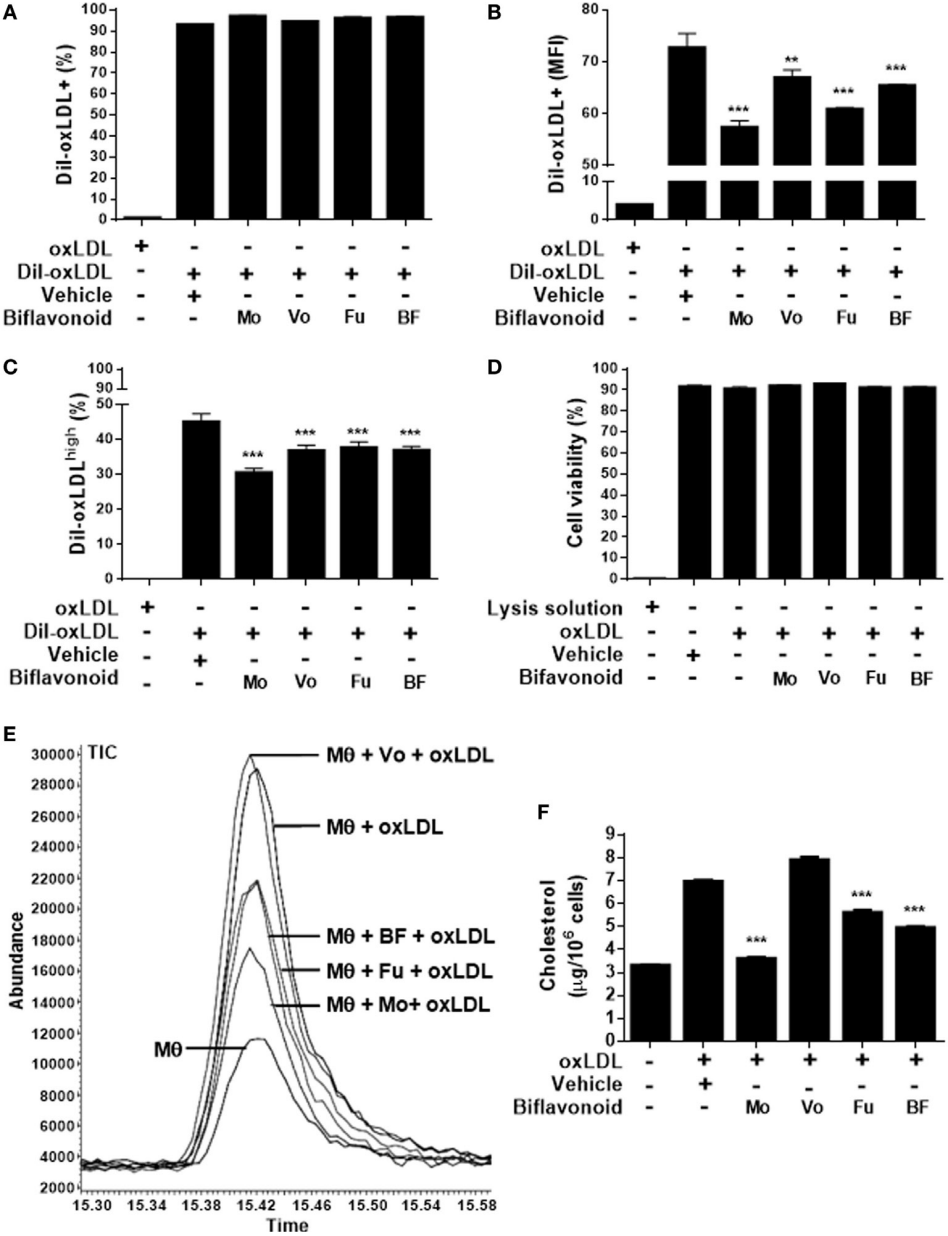
Biflavonoids modulate oxidized LDL (oxLDL) uptake and cholesterol accumulation in primary macrophages. Macrophages were preincubated 12 h with biflavonoids (45 µM for Mo, Vo, and Fu, and 25 µg/mL for BF) and subsequently incubated with DiI-oxLDL (50 µg/mL) for additional 6 h. Labeled oxLDL–macrophage uptake was then determined by flow cytometry and graphed as the percentage of DiI-oxLDL^+^ cells **(A)**, the MFI in DiI-oxLDL^+^ cells **(B)**, or the percentage of DiI-oxLDL^high^ cells **(C)** based on a gating strategy shown in the Figure S2B in Supplementary Material. Under the experimental conditions used in these assays, macrophage viability was preserved as shown by LDH release into cell supernatants **(D)**. Parallel biflavonoid-pretreated macrophage cultures (12 h) were further incubated in the presence of oxLDL (30 µg/mL) during 36 h and the cellular cholesterol content was determined by GC/MS; a representative chromatogram is shown **(E)**. Cholesterol content was calculated based on the peak area of the ion 145 [for details of the method, see Ref. (23)] and expressed as microgram cholesterol/10^6^ macrophages **(F)**. Experiments were performed in triplicate and bars represent the mean ± SD. **p < 0.01, ***p < 0.001, compared to vehicle-treated cells. The results are representative of at least three independent experiments. Mθ: macrophage.

Growing evidence suggest that the direct antioxidant effects of flavonoids are insufficient to explain their atheroprotective function *in vivo* and that other mechanisms down stream might be more important when their dietary use is considered (13). A key step in the “atherogenic pathway” is the uncontrolled surface upregulation of the CD36 SR in response to the proatherogenic ligand oxLDL, leading to its massive uptake and the subsequent progressive transformation of macrophages into foam cells (28). Thus, we tested whether biflavonoids interfere with this process. Conditions (time and concentration of biflavonoids and oxLDL) used for macrophage treatments were previously set to assure the preservation of cell viability in the assays (Figure S1 in Supplementary Material). Flow cytometry histograms showed that in the basal state most of macrophages express CD36 at high density on the surface, which is further increased after exposure to oxLDL [Figure S2A in Supplementary Material (23)]. Therefore, it was not surprising that treatments with biflavonoids did not alter the percentage of the CD36^+^ cells (Figure 3A; Figure S2A in Supplementary Material). Interestingly, the surface density of the CD36 receptor (as determined by the MFI) or the frequency of CD36^high^ macrophages was significantly reduced by biflavonoid treatment (Figures 3B,C). In the absence of oxLDL triggering, no changes were induced on the surface density of CD36 by biflavonoid treatment (Figure 3D), indicating that biflavonoids are active only when macrophages are acquiring a proatherogenic phenotype. The consequences of this biflavonoid-mediated reduction of CD36 expression on macrophages were tested by examining their transformation into foam cells using the oxLDL uptake assay and by quantifying the accumulation of intracellular cholesterol. Results of fluorescently labeled oxLDL uptake, both in percentages as well as in MFI (which reveals the magnitude of oxLDL loading) closely mirrored those of CD36 expression (Figures 3A–C and 4A–C; Figure S2 in Supplementary Material). Moreover, macrophages treated with Mo or Fu accumulated less cholesterol intracellularly than vehicle-treated cells (Figures 4E,F). Taken together, results indicate that biflavonoids from *G. madruno* are not only able to reduce the formation of the proatherogenic ligand oxLDL but also to downregulate its cognate endocytic receptor on macrophages, resulting in the attenuation of modified lipoprotein uptake and foam cell formation.

### ROS Production and Proinflammatory Cytokine Secretion in Proatherogenic Macrophages Are Also Inhibited by Biflavonoids

**Figure 5:**
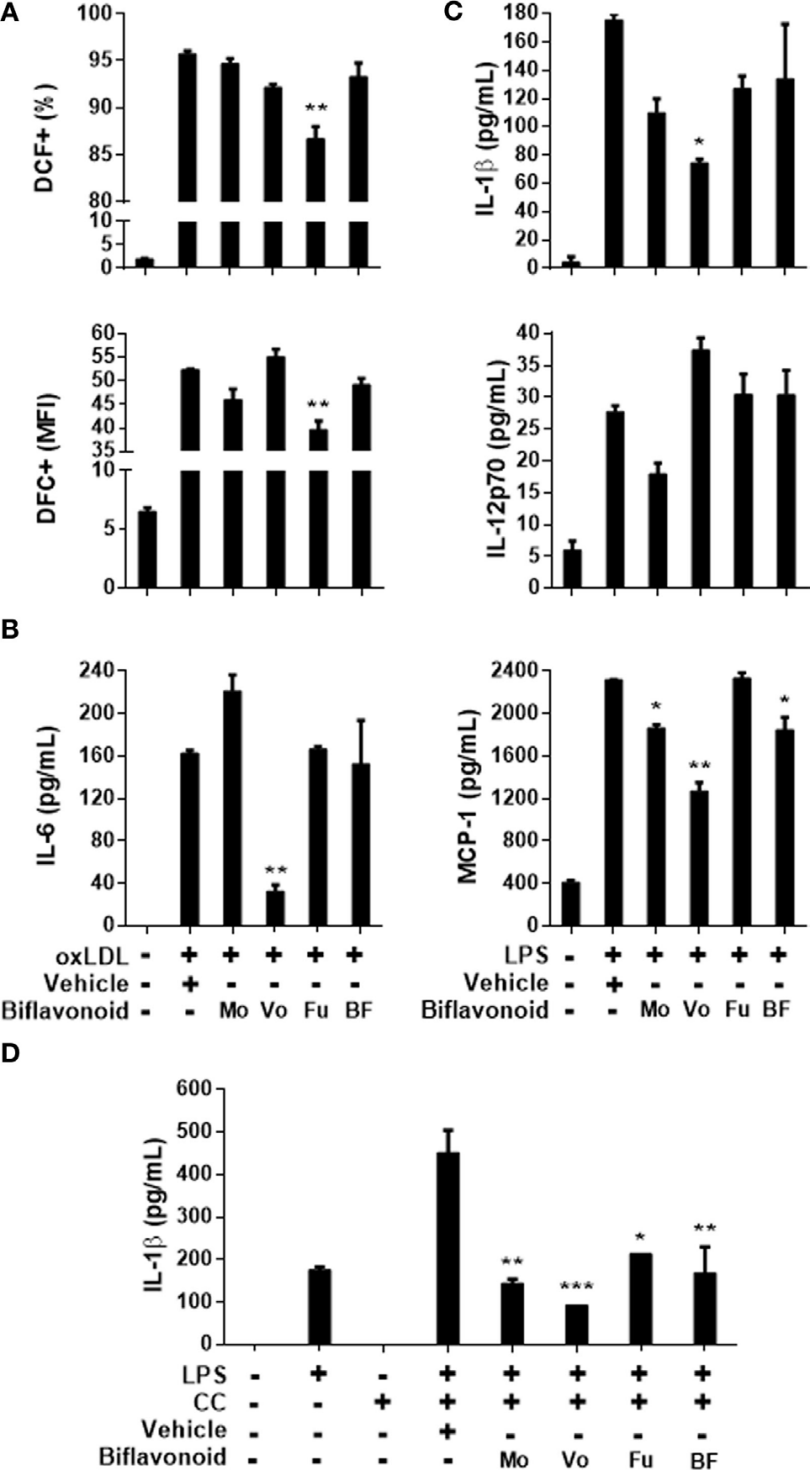
Reactive oxygen species (ROS) and proinflammatory cytokine responses of macrophages to oxidized LDL (oxLDL), lipopolysaccharide (LPS), and crystal stimulation are modulated by biflavonoids. Biflavonoid-pretreated macrophages [12 h; 90 µM for Mo, Vo, and Fu, and 50 µg/mL for BF] were further incubated with oxLDL (1 h, 50 µg/mL) and ROS production was subsequently monitored with the DFC probe by flow cytometry; results are shown as the percentage and MFI of DFC^+^ cells **(A)**. Biflavonoid-pretreated macrophages (12 h; 45 µM for Mo, Vo, and Fu, and 25 µg/mL for BF) were also stimulated for additional 12 h with oxLDL (25 µg/mL) in order to quantify IL-6 secretion **(B)**, or with LPS (10 µg/mL) to quantify the secretion of IL-1β, IL-12p70, and MCP-1 **(C)** into culture supernatants. IL-1β release to culture supernatants was also assessed in biflavonoid-pretreated cells (12 h; 45 µM for Mo, Vo, and Fu, and 25 µg/mL for BF) that were subsequently LPS-primed (10 ng/mL) and cholesterol crystal (500 μg/mL)-stimulated **(D)**. Cytokine quantifications were performed by Luminex. Results are graphed as the mean ± SD from triplicate cell cultures. *p < 0.05, **p < 0.01, ***p < 0.001, compared to vehicle-treated cells. The results are representative of at least three independent experiments. None of the treatments altered cell viability as determined by LDH release assay (not shown).

Besides foam cell formation, triggering of oxLDL receptors on macrophages leads to profound and complex cellular immunometabolic changes that appear to be essential for atherogenesis. *In vitro* and *in vivo* evidence indicate that the ROS production and the secretion of proinflammatory cytokines are crucial components of such a response (4). When the highly sensitive probe DCFH-DA was used to quantify ROS production in oxLDL-stimulated macrophages by flow cytometry, a significant reduction in the frequency of DCF^+^ cells and the MFI was observed in cells pretreated with the biflavonoid Fu (Figure 5A). Mo and Vo also inhibited macrophage pro-oxidative response at some extent, but differences with vehicle-treated cells did not reach statistic significance. We next examined the ability of biflavonoids to modulate cytokine secretion in macrophage cultures. First, we tested the effects of biflavonoids in resting macrophages and no significant proinflammatory activity was evidenced (Figure S3 in Supplementary Material). Then, we evaluated the effects of biflavonoids on the oxLDL-stimulated IL-6 production, since, under our experimental conditions, this cytokine is the only one induced by oxLDL in macrophages (23). Vo but no other biflavonoids mediated a significant inhibition (Figure 5B). Because TLR4 triggers a pathophysiologically relevant pathway in atherosclerosis (29), we also determined whether biflavonoids alter the cytokine response to the ligand LPS. We found that all cytokines tested were robustly upregulated in response to LPS and that all aglycone biflavonoids inhibit one or more of the cytokines IL-1β, IL-12p70, and MCP-1 with a particular signature (Figure 5C). Interestingly, the morelloflavone glycoside Fu was devoid of this anti-inflammatory effect. No changes were observed in the levels of IL-6, TNFα, or MIP-1α. Finally, given the recognized proinflammatory role of cholesterol crystals during atherosclerosis *via* nucleotide-binding domain and leucine-rich repeat containing protein 3 (NLRP3) sensing and assembly (30) we analyzed the effect of biflavonoids on cholesterol crystal-induced IL-1β secretion using low-dose LPS-primed macrophages, as reported previously (23). All biflavonoids were inhibitory in this system (Figure 5D), suggesting a potential modulatory effect of this group of molecules on NLRP3 inflammasome activity. Collectively, our findings confirm that biflavonoids extend their antiatherogenic activities by acting on effector proinflammatory mediators, such as ROS and cytokines, produced by macrophages in response to an array of proatherogenic signals.

### A Defined Biflavonoid Fraction (BF) Is Atheroprotective *In Vitro* and *In Vivo*

**Figure 6:**
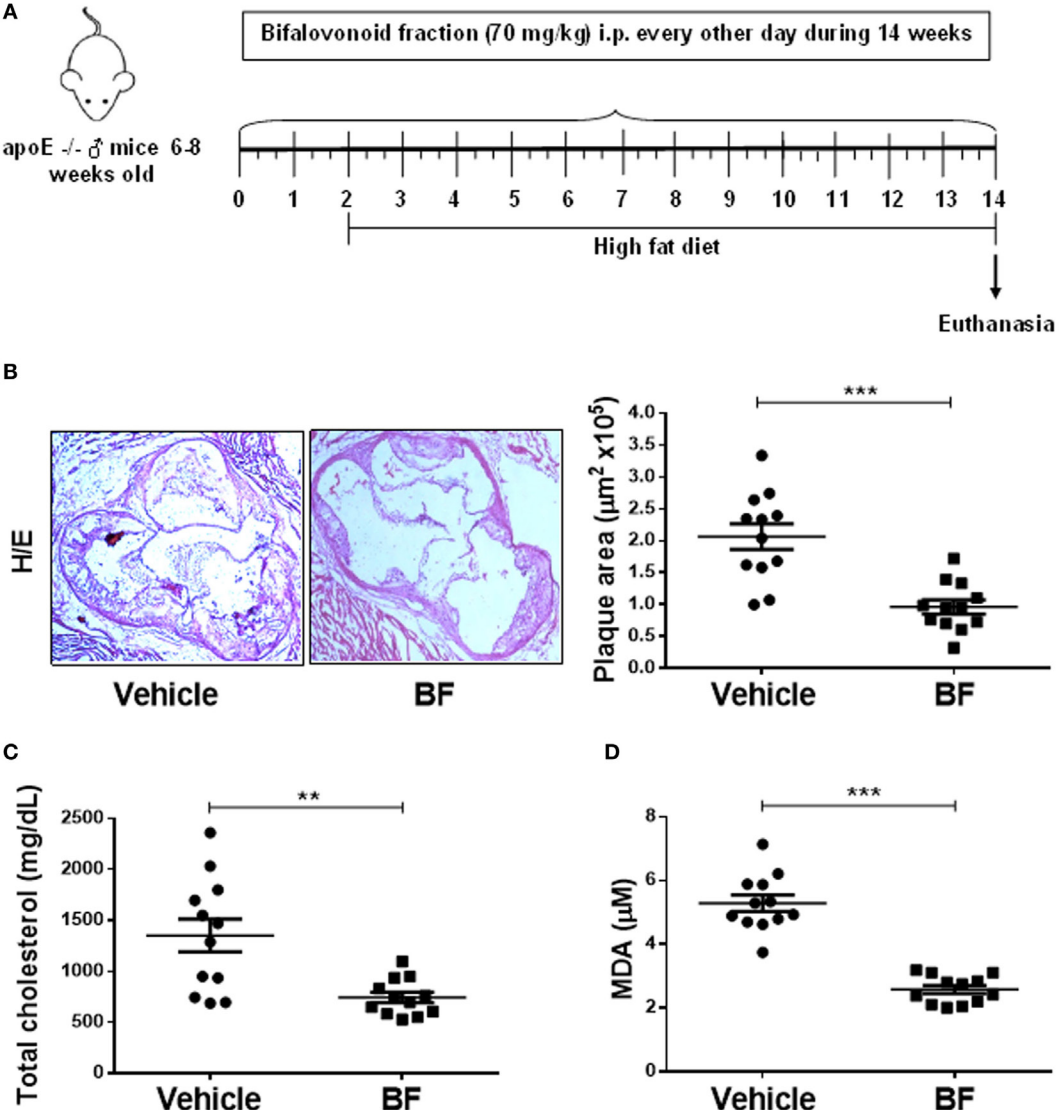
A *Garcinia madruno*’s BF is atheroprotective *in vivo*. ApoE^−/−^ mice were treated i.p. with the BF (70 mg/kg, every other day) and fed a western diet, as shown **(A)**. Vehicle (DMSO 0.1% in PBS)-treated mice were used as negative controls. After 14 weeks of treatment, sections of the aortic sinus were stained with H/E. Representative microphotographs for each treatment group are shown [**(B)**, left]. The size of atherosclerotic lesions was calculated using an image analysis software (see Materials and Methods for details). Each point represents the average lesion area (out of 9–12 sections) per mouse [**(B)**, right]. Serum samples from same animals were used to quantify total cholesterol levels **(C)** and MDA concentration **(D)**. Data from individual mice are presented and bars correspond to the mean ± SEM. **p < 0.01, ***p < 0.001, compared to control group (Mann–Whitney *U* test). These results are representative of two independent experiments.

**Figure 7:**
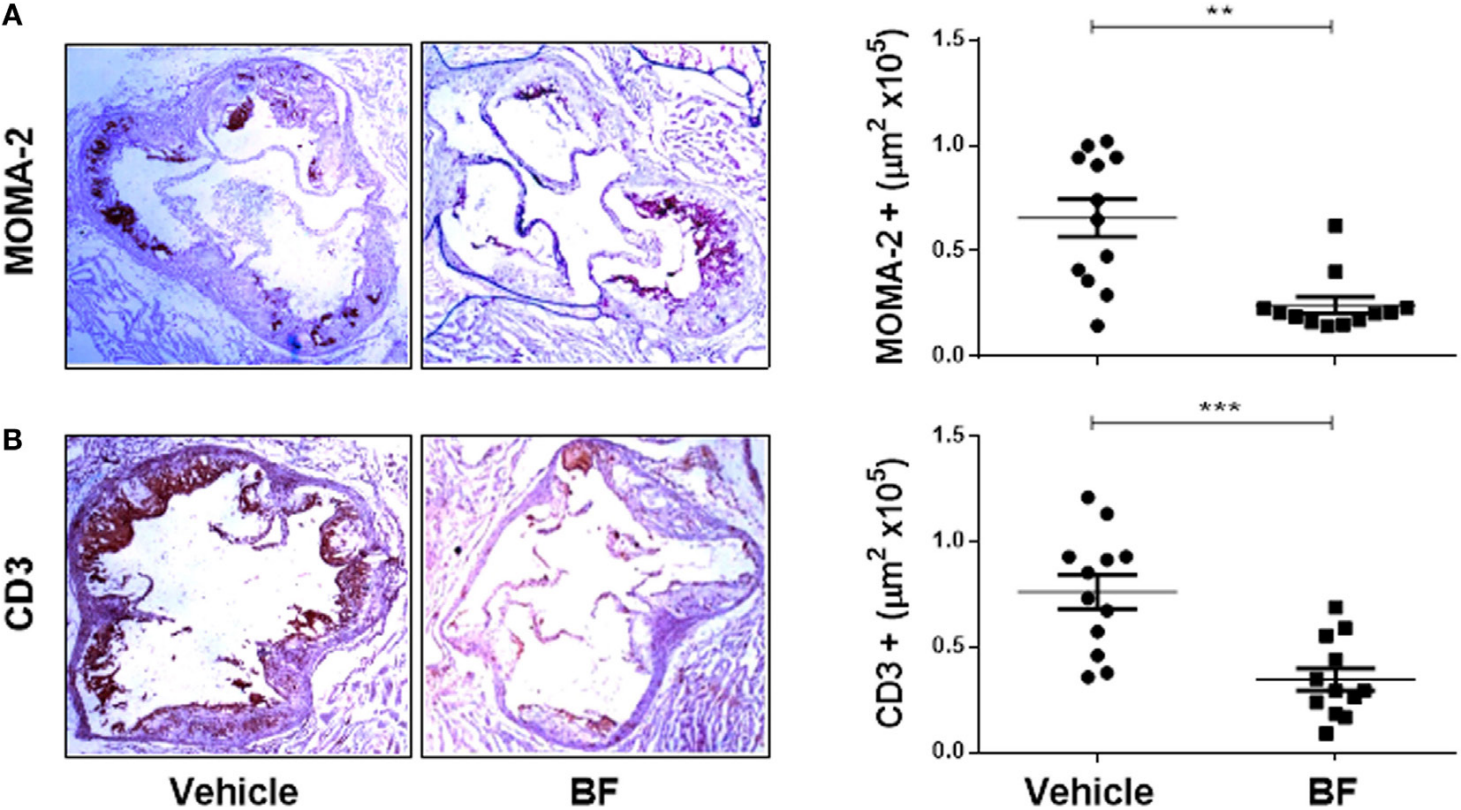
*In vivo* atheroprotective effect of the *Garcinia madruno*’s BF is related to reduced macrophage and T cell infiltration in the atherosclerotic lesions. ApoE^−/−^ mice were treated i.p. with the BF and fed a high-fat diet, as shown in Figure 6A. A group of mice treated with vehicle (DMSO 0.1% in PBS) were used as controls. After 14 weeks, sections of the aortic sinus were immunostained with macrophage-(MOMA-2) **(A)** or T cell-(CD3) **(B)** specific antibodies. Representative microphotographs for each treatment group are shown (left). The leukocyte infiltrate in the atherosclerotic lesions was calculated using an image analysis software (see Materials and Methods for details). Each point represents the average positive area (out of 9–12 sections) per mouse (right). Data from individual mice are presented and bars correspond to the mean ± SEM. **p < 0.01, ***p < 0.001, compared to control group (Mann–Whitney *U* test). These results are representative of two independent experiments.

Having demonstrated that biflavonoids inhibit several relevant atherogenic events at the oxLDL–macrophage interphase *in vitro*, our next question was whether biflavonoids are atheroprotective *in vivo*. Given the large amounts of biflavonoids required, a BF from *G. madruno* with a defined chemical composition (as described in the Section “Materials and Methods”) was prepared for these experiments. First, we confirmed that this biflavonoid mix preserved the *in vitro* properties of its major components Mo and Vo. As shown in Figures 2–5, the BF inhibited LDL oxidation (Figures 2C–E), CD36 surface expression (Figures 3B,C), oxLDL uptake (Figures 4B,C), and cholesterol accumulation (Figures 4E,F), with a potency that reflects its biflavonoids composition. Similarly, just like Mo and Vo, the BF was inactive in the macrophage ROS production inhibition assay (Figure 5A), and in the cytokine response assays to cholesterol crystals, oxLDL, or LPS, the presence of BF in cell cultures also reflected its biflavonoid composition (Figures 5B–D). Next, we used ApoE^−/−^ mice to evaluate the atheroprotective effect of BF administered using a route of administration (i.p.) and a scheme (every other day during the whole experiment; Figure 6A) that is expected to improve the systemic exposure to the active flavonoid aglycones (31). Figure 6B shows that, compared to vehicle-treated mice, BF-treated mice had significantly smaller atheromatous lesions in the aortic sinus (46% reduction). The atheroprotective effect of BF at the given dose and scheme was observed in the absence of any sign of toxicity, as determined by monitoring of whole body weight (Figure S4A in Supplementary Material) or other parameters, such as food intake, physical appearance, hydration, or behavior, during the experiment (not shown). Biochemical hepatotoxicity or pancreatic toxicities were also ruled out, since blood levels of hepatic enzymes (AST and ALT) or amylases were no modified by BF treatment (Figures S4B–D in Supplementary Material). Gross macroscopic appearance of internal organs, such as liver, kidneys, spleen, and heart, were similar in treated and control mice. Interestingly, serum cholesterol levels, as well as the levels of the oxidative stress marker MDA, were lower in BF-treated mice (Figures 6C,D), indicating metabolic-modifying and oxidation-protective effects of biflavonoids *in vivo*. En face aortic OR^+^ areas in BF-treated mice showed a trend to lower lipid deposition as compared to vehicle-treated mice; however, differences were not significant (Figure S5 in Supplementary Material). Finally, the effect of BF treatment on the local macrophage and T cell inflammatory infiltrate was determined by immunohistochemical assessment of MOMA-2 and CD3 expression, respectively, in aortic sinus sections. Macrophage and T cell infiltrates were significantly lower in BF-treated mice (63 and 55% reduction, respectively; Figures 7A,B), which is consistent with the reduction of atheroantigen formation/accumulation, reduced foam cell formation, and reduced inflammatory response observed *in vitro* (Figures 2D,E, 4 and 5), demonstrating that natural biflavonoids are immunomodulatory *in vivo*. Collectively, these results are in agreement with the *in vitro* effects described in previous sections, and further confirm that biflavonoids have hypolipemiant, antioxidant, immunomodulatory, and atheroprotective activity *in vivo*.

## Discussion

A large body of experimental evidence supports the notion that the interaction between macrophages and oxidatively modified LDL dictates pathological events in the arterial wall that result in the continuous and progressive development of atheromatous lesions (4); therefore, atherogenic macrophages are considered key therapeutic targets. Flavonoids have been shown to alter several aspects of macrophage physiology (23, 32) in a manner that suggest that, at least part of their atheroprotective properties, are mediated *via* this cell type. Here, we show that biflavonoids, a more restricted class of naturally occurring phytochemicals, inhibit the proatherogenic response of macrophages to oxLDL *in vitro* and attenuate atherosclerotic lesion development *in vivo*. Our results expand the understanding of the pharmacology of these compounds and stimulate further exploration of their use for atherosclerosis and other macrophage-related disorders.

Many studies *in vitro* and *in vivo* animal models as well as human epidemiological and clinical studies support the theory that, under hypercholesterolemic conditions, LDL transformation into oxLDL in the vascular subintimal space triggers primary fatty strake formation and subsequent atherosclerotic plaque development (33, 34). Retained LDL particles are exposed to ROS generated in the arterial wall *via* several oxidative mechanisms (35), leading to complex chemical processes that result in the formation of oxidatively modified LDL particles, or oxLDL. oxLDLs are highly heterogeneous and contain an array of lipid peroxidation products in the lipidic component that are responsible of many down stream damaging effects on vascular cells, such as cell death, proliferation, migration, and inflammatory response (33, 34). *In situ* oxidation also generates modified derivatives and fragments of the apolipoprotein component ApoB-100, which function as modified self-ligands triggering foam cell transformation and further proinflammatory response *via* innate receptor recognition (34, 36). Moreover, modified protein components in oxLDL behave as neoantigens for the adaptive immune response *via* T-cell receptor and B-cell receptor stimulation on T and B lymphocytes (36), whose proinflammatory effector responses are critical in the chronic phase of the disease. It is, therefore, reasonable that dietary or pharmacological prevention of LDL oxidation represents an interesting intervention strategy, since reduced availability of the atherogenic ligand oxLDL would be translated into less lipid accumulation, decreased innate and adaptive inflammatory responses, and reduced overall damaging effects. We found that biflavonoids were as potent as monoflavonoids to scavenge ROS (Figures 2A,B), and most importantly, protected LDL from Cu^2+^-induced lipid oxidation (Figure 2C), demonstrating that in flavonoid dimers this antioxidant activity is preserved. Biflavonoids not only reduced the production of lipid peroxides but also prevented structural changes (such as increased electronegativity) in the LDL’s apolipoprotein component (Figures 2D,E). Our results are consistent with previous work reporting free radical scavenging effects of biflavonoids and biflavonoid-containing preparations in several systems (15, 17, 21, 22), and extend them by showing, for the first time, the protective effects of Mo, Vo, and Fu against ApoB-100 modification. Interestingly, the biflavonoid Mo and its glucoside Fu were comparable in their ability to protect LDL, whereas Vo was a weaker protector of lipoprotein modification. Considering the variety of chemical species formed in the oxLDL particle, these observations might be explained by qualitative/quantitatively different effects of particular biflavonoids on different reactive species. This is of physiological relevance, since relative amounts of different chemical species are thought to explain the documented heterogeneity, and even duality, of oxLDL cellular effects [e.g., induction of proliferation/migration versus cell growth arrest/apoptosis (37)]. Thus, differential effects of biflavonoids on oxLDL composition might impact differently the extent and spectrum of potential atherogenic effects of oxLDL on vascular cells, such as EC, SMC, or macrophages. Studies addressing the effects of biflavonoid-treated oxLDL on those cell types will allow to confirm this hypothesis.

Although a key consequence of the increasing atherogenic ligand availability in the intimal space is the progressive alteration of vascular SMC and EC functions, perhaps the most prominent proatherogenic effect of oxLDL is the promotion of macrophage to foam cell transformation. This phenomenon critically depends on the expression of oxLDL receptors on macrophages, and their intracellular signal transduction. Canonical foam cell formation results from the uncontrolled oxLDL uptake by resident and monocyte-derived immigrant macrophages *via* SR, such as CD36 (28). “Trapping” of foamy macrophages in the intima is also CD36-mediated (28), further emphasizing the importance of this receptor in the atherosclerotic process. All biflavonoids tested here were effective at reducing oxLDL-triggered CD36 overexpression on macrophages and the subsequent intracellular accumulation of cholesterol and transformation into foam cells (Figures 3 and 4). Our results are the first to document such effect for biflavonoids and are consistent with reports showing similar anti-foam cell formation effects exerted by several phytochemicals, including aglycone monoflavonoids, such as Quercetin and Kaempferol (23, 38–41), glycoside monoflavonoids (42), and glucuronide metabolites (43). The molecular mechanisms underlying the CD36 downregulatory effect reported here are yet to be investigated. Preliminar evidence with monoflavonoids and other phytochemicals points to PPARγ antagonistic effects (38, 42) and inhibition of c-Jun-activating protein-1 (AP-1) nuclear translocation (40), but further characterization is required. Results of LDL uptake paralleled those of CD36 expression, suggesting that, in our system, most of oxLDL internalization occurred *via* this receptor. However, since macrophages also internalize oxLDL *via* SR-A, we cannot rule out a possible role for this SR (44). Whereas SR-A does not appear to be downregulated by monoflavonoid treatment (40), future studies should evaluate whether, besides CD36, biflavonoids also inhibits SR-A expression in macrophages. Net cholesterol accumulation in macrophages is influenced by two opposite processes, namely influx from oxLDL *via* SR and efflux *via* ATP-binding cassette (ABC) transporters (28, 44). Whether biflavonoids increase ABC transporters expression and, consequently, cholesterol efflux contributing to reduced foam cell transformation was not investigated here, but it appears as an interesting possibility that requires further testing. This mechanism has gained recent attention since many monoflavonoids improved reverse cholesterol transport through the upregulation of ABC transporters and subsequent increase of cholesterol efflux (45–47). Thus, as monoflavonoids appear to inhibit foam cell formation through reduced oxLDL uptake and increased cholesterol efflux, we speculate that biflavonoids, either directly or after their breakdown into monoflavonoids, exert similar activities. In support of this hypothesis, a recent report documented that Apigenin, one of Vo monoflavonoids, promoted cholesterol efflux in macrophages (48). Altogether, our results so far indicate that biflavonoids reduce both athero-ligand formation and athero-receptor expression, which configurate a powerful mechanism for targeting the anatomic and functional hallmark of atherosclerosis: the foam cell.

Macrophage recognition of oxLDL and other atherogenic ligands triggers, through CD36 and other innate receptors, a powerful inflammatory response that fuels and amplifies the atherogenic process. The production of ROS and the secretion of proinflammatory cytokines make an integral part of this response (49). We report here for the first time that Mo, Vo, and Fu inhibit oxLDL- or LPS-induced ROS production or proinflammatory cytokine secretion in macrophages, with a particular pattern (Figures 5A–C). For instance, the glucoside Fu was a weak/inactive inhibitor of cytokine secretion but effective inhibitor of ROS production, whereas the aglycones Mo and Vo exhibited the opposite trend. Among aglycones, Mo was the most active inhibiting the Th1-inducing cytokine IL-12, whereas Vo was the strongest attenuator of IL-6, MCP-1, and IL-1β production. The proatherogenic roles of macrophage-derived ROS and those cytokines are well documented and includes the promotion of chemotaxis, local/systemic inflammation, T cell activation, apoptosis, and necrosis (49, 50), pointing up to the potential antiatherogenic mechanisms of *G. madruno*’s biflavonoids. A number of biflavonoid preparations have been shown to inhibit or downregulate enzymes involved in the production of bioactive lipids in macrophages, such as phospholipases, cyclooxygenases, and lipoxygenases (16). Moreover, some biflavonoids inhibited the expression of inducible nitric oxide synthase (iNOS) (and subsequent nitric oxide production), cyclooxygenase 2 (COX-2) (and subsequent production of lipid mediators, such as prostaglandin E2, PGE_2_), TNFα and other proinflammatory cytokines in macrophages (16, 51–54). Those studies, however, were mostly performed in macrophage cell lines and generally used LPS as a sole stimulus. Our study extends the knowledge to primary macrophages with the non-previously tested biflavonoids Mo, Vo, and Fu, exploring ROS and cytokine production in the context of oxLDL- in addition to LPS-stimulation. The signaling pathways involved in the inhibitory effect reported here are yet to be investigated. However, taking in consideration the reports implicating ERK/c-Fos-AP-1 and ERK/nuclear factor-κB as key pathways mediating the iNOS/COX-2-inhibitory effects of the biflavonoids Am, Ochnaflavone, and a Ginkgo Biloba extract (53, 55, 56), we propose that these pathways could play an important role. Again, the plethora of signaling pathways and transcription factors affected by monoflavonoids (57, 58) represent a collection of potential targets for biflavonoids and their metabolites that will be revealed in the coming years. An interesting recent report showed that the biflavonoid mix kolaviron reduced ROS and proinflammatory cytokines in LPS-stimulated microglial cells and this effect was dependent on the nuclear factor erythroid 2-related factor 2/antioxidant responsive element [Nrf-2/antioxidant responsive element (ARE)] pathway (59). Nrf-2/ARE has been emerging as a key indirect antioxidant and anti-inflammatory pathway triggered by phytochemicals, such as flavonoids, with relevance in various inflammatory diseases (60, 61), including atherosclerosis (62). Further investigations with other biflavonoids are assured.

Interleukin-1β is a proinflammatory cytokine with increasing pathophysiological and therapeutic relevance in atherosclerosis and, more generally, in sterile inflammation (30, 63). Basic research indicates that oxLDL mediates a CD36-dependent endocytic pathway leading to ligand accumulation, cholesterol nucleation, and crystal formation (64), as well as pro-IL-1β production (65). Later, cholesterol crystals promote lysosomal disruption, NLRP3 inflammasome activation and subsequent IL-1β maturation *via* caspase-1-dependent proteolysis (66). Being a key “upstream” inflammatory mediator in CVD (67), the targeting of IL-1β actions with anti-cytokine or anti-receptor mAb has emerged as a promising therapeutic option (67, 68). Moreover, preventing IL-1β production with NLRP3 small molecule inhibitors has also gained recent attention (69). When we assessed the effect of biflavonoids on cholesterol crystal-induced IL-1β secretion by macrophages, we found a significant inhibitory effect (Figure 5D), suggesting that biflavonoids might act as NLRP3 inflammasome inhibitors. Future experimental settings that allow separating a possible NLRP3-inhibitory effect (signal 2) from the pre-IL-1β downregulatory activity (signal 1) will permit the confirmation of this hypothesis. In support of this, a similar suggestive observation was made with the monoflavonoid quercetin (23), which was recently confirmed as a direct inhibitor of the NLRP3 inflammasome activation *via* interference with the oligomerization of the adaptor protein apoptosis-associated speck-like protein containing a CARD (70). Phytochemicals, such as flavonoids, are typically effective in diseases where IL-1β production and NLRP3 inflammasome activation make part of the pathophysiology, and growing evidence support the action of these compounds on NLRP3 activation (71). Thus, biflavonoids/monoflavonoids could represent a novel class of small molecule NLRP3 inflammasome inhibitors.

Consistent with the *in vitro* atheroprotective effects of biflavonoids in macrophages, i.p. administration of the BF attenuated atherosclerosis development *in vivo*, and this effect was accompanied by less oxidative stress, a reduction in macrophages and T cells infiltrating atherogenic plaques and by lower plasma cholesterol levels (Figures 6 and 7). Although a causal relationship remains to be established, we propose that the antiatherogenic effects of BF evidenced in primary macrophages *in vitro* also operate *in vivo* and explain, at least in part, the effects of BF in dyslipidemic mice. Pinkaew and cols reported that diet supplementation with Mo, the majority biflavonoid in our BF, also induced significant atheroprotection (18) that was associated with reduced migration of SMC to the intimal space but not with reduced oxidative stress, hypocholesterolemic effects, or changes in macrophage density in the aortic sinus. Discrepancies between the two studies could be explained by different experimental settings, such as different genetic background of the animals, oral versus i.p. administration, chow versus HFD diets, different duration of treatments, or different composition of the biflavonoid preparation. It is possible that biflavonoids exert atheroprotection *via* both SMC and macrophage-intrinsic effects. Moreover, the observation that Mo inhibits angiogenesis *in vitro* and *in vivo* (19) makes tempting to propose that atheroprotective actions of this biflavonoid also involve the third key cell in the vascular wall, the EC. Mechanistic studies have indicated that Mo alters SMC and EC migration by targeting migration-related kinases, such as focal adhesión kinase, s-Src and ERK, and small GTPases, such as RhoA and Rac1 (19, 20). It will be interesting to confirm whether Mo also alters macrophage migration and whether similar signaling pathways are inhibited in proatherogenic macrophages.

Although very little is known on the bioavailability and biotransformation of biflavonoids (16, 17), they surely follow the same rules of monoflavonoids, this is, short half-life, rapid conjugation and excretion, and intestinal transformation by microorganisms. The reduced anti-inflammatory activity *in vivo* upon oral administration compared to i.p. administration is likely the result of the poor oral bioavailability of biflavonoids and/or active biflavonoid metabolites (16). One study reported that after oral Mo supplementation (200 mg/kg/day) in mice, blood levels of Mo were almost indetectable [1.37 µM; (20)]. Although measurements of the serum levels or tissue distribution of biflavonoids were not performed in our work, we chose chronic i.p. administration (70 mg/kg/every other day) in order to assure systemic exposure to biflavonoid or their potentially active metabolites during the whole experiment. This strategy allowed evidencing the antioxidant, hypocholesterolemic and anti-inflammatory effects of the BF (Figures 6 and 7). In Pinkaew’s atheroprotection study, a very low dose of oral Mo (4 mg/kg/day) that is expected to yield very low (submicromolar range) serum concentrations (18) was used. However, reduced SMC migration and significant atheroprotection (as assessed by atheromatous plaque size) was observed, in the absence of hypocholesterolemic, antioxidant, or anti-inflammatory effects. As suggested by the study where Mo inhibited directly the HMG-CoA Reductase (72), a hypocholesterolemic effect of Mo would require systemic concentrations hardly reached by oral low-dose administration but possibly reached after parenteral injections (73). Likewise, *in vivo* antioxidant and anti-inflammatory effects might require higher circulating levels of Mo than those reported/expected in Pinkaew’s studies (18, 20). On the bases of the recent hypothesis of a “xenobiotic-like” effect of flavonoids (13), it is possible that lower MDA levels and reduced inflammatory infiltrate observed in our work were the result of “indirect” antioxidant and anti-inflammatory effects promoted by biflavonoid actions on the Nrf-2/ARE axis (59–62). The extent of these effects and, therefore, the range of atherogenic processes and cells that are targeted by biflavonoids *in vivo* could be influenced by factors such as dose/scheme/*via* of administration or purity/complexity of the biflavonoid preparation (18–20). Adding more complexity to the biflavonoid’s pharmacology, it is currently unknown whether biological effects of biflavonoids are due to their breakdown into monoflavonoids or to intrinsic structural features. In favor of the first hypothesis is the observation that biological/pharmacological characteristics of biflavonoids and monoflavonoids are largely overlapping. For instance, monoflavonoids conforming the biflavonoids reported here, such as naringenin, apigenin, or luteolin are also endowed with anti-foam cell, antioxidant/anti-inflammatory and/or atheroprotective properties (16, 45, 47, 57, 58). However, evidence also argues toward the alternative hypothesis, since some activities of BF are not reproduced with their monoflavonoid counterparts (74, 75). Furthermore, as shown with dimeric (and polymeric) procyanidins (76), an array of active metabolites can be generated by microbial decomposition of mono and biflavonoids to phenolic acids. Thus, activities of biflavonoids might well result from a complex combination of: (i) direct effects of the biflavonoid and conjugated biflavonoids, (ii) effects of the conjugated/unconjugated biflavonoid-derived monoflavonoids, and (iii) effects of the conjugated/unconjugated microbial-derived decomposition products. This scenario widens the spectrum of potential targets and makes the understanding of biflavonoid activities even more challenging.

In summary, we have shown information demonstrating the atheroprotective activity *in vivo* and *in vitro* macrophage-intrinsic effects for biflavonoids. Improving our understanding of the mechanisms of action of these phytochemicals will increase their preventive and therapeutic potential for cardiovascular and other inflammatory diseases where macrophages and macrophage-related cells play pathogenic and/or protective roles.

## Ethics Statement

This study was carried out according to the guidelines for the care and use of laboratory animals; all of the procedures were approved by the institutional animal care committee (Comite institucional para el cuidado y uso de los animals, Universidad de Antioquia).

## Author Contributions

JT-G, OL-G, and JR-P were responsible for conception and design of the study. JT-G and OL-G performed experiments. EO prepared pure biflavonoids and BF. RA performed GC/MS cholesterol quantifications. YL-V helped with *in vivo* experiments. JS and JL-L provided material and/or methodological input. JT-G, OL-G, and JR-P analyzed results and interpreted data. JR-P supervised the study. JT-G and JR-P wrote the manuscript. All authors read and approved the final manuscript.

## Conflict of Interest Statement

The authors declare that the research was conducted in the absence of any commercial or financial relationships that could be construed as a potential conflict of interest.

## Supplementary Material

Figure S1 Viability of macrophage cultures in response to oxLDL or biflavonoids. Macrophages were treated during the indicated time and with the indicated amounts of oxLDL **(A)** or biflavonoid preparations **(B)** to determine cell viability by measuring LDH activity in the supernatants. Vehicle and a cell lysis solution were used as controls. Experiments were performed in triplicate, and bars represent the mean ± SD.

Figure S2 Flow cytometry analysis of CD36 surface expression and oxLDL uptake by macrophages after treatments. The effect of biflavonoids (here only Mo is shown as a representative example) on CD36 expression **(A)** or oxLDL uptake **(B)** in macrophages was investigated by flow cytometry (as explained in Materials and Methods). Representative histograms for the indicated condition are shown. Controls for CD36 expression under resting [**(A)**, left] or oxLDL-stimulation conditions [**(A)**, middle] are shown for comparison. Controls for non-fluorescent [**(B)**, left] or fluorescent oxLDL (DiI-oxLDL) uptake [**(B)**, middle] are also shown for comparison. Bars on the histograms indicate CD36^+^ and CD36^high^ macrophages as well as DiI-oxLDL^+^ and DiI-oxLDL^high^ macrophages. I: isotype controls. Mθ, macrophages.

Figure S3 Effect of biflavonoids on proinflammatory cytokine production in resting macrophages. Macrophages were treated with biflavonoids (24 h; 45 µM for Mo, Vo, and Fu, and 25 µg/mL for BF) or vehicle and the secretion of proinflammatory cytokines IL-6 **(A)**, IL-1β **(B)**, TNFα **(C)**, MCP-1 **(D)**, MIP-1α **(E)**, and IL-12p70 **(F)** was evaluated by Luminex. LPS (10 μg/mL)-treated macrophages were used as positive control. Experiment was performed in triplicate and bars represent the mean ± SD.

Figure S4 Treatment with *Garcinia madruno*’s BF does not alter mouse body weight or clinical biochemistry parameters. ApoE^−/−^ mice were treated i.p. with the biflavonoid fraction (70 µg/kg), as shown in Figure 6A. Total body weight was monitored weekly and compared to vehicle-treated mice **(A)**. Serum samples were obtained from animals at the end of treatments and the levels of aspartate transaminase (AST) **(B)**, alanine transaminase (ALT) **(C)**, and amylase **(D)** were determined. Body weight results are shown as the mean ± SEM, whereas biochemical parameters of mice are shown individually with bars indicating the mean ± SEM. No significant differences were found between the two groups. Dotted lines represent normal reference ranges for each analyte.

Figure S5 Evaluation of the effect of BF treatment on aortic lipidic deposition in ApoE^−/−^ mice. ApoE^−/−^ mice were treated i.p. with the biflavonoid fraction (70 µg/kg), as shown in Figure 6A. Vehicle-treated mice were used as negative controls. After sacrifice, aortas (from ascendant to abdominal) were removed, opened longitudinally, and stained with Oil Red O. Oil Red^+^ positive areas were calculated with the aid of image analysis software. Results are shown individually with bars indicating the mean ± SEM. No significant differences were found between the two groups.

## Abbreviations

ABC, ATP-binding cassette; ALT, alanine transaminase; Am, amenoflavone; AP-1, activating protein-1; ApoB-100, apolipoprotein B-100; ApoE^−/−^, apolipoprotein E-deficient; ASC, apoptosis-associated speck-like protein containing a CARD; AST, aspartate transaminase; ARE, antioxidant responsive element; BCR, B-cell receptor; BF, biflavonoid fraction; CC, column chromatography; COX-2, cyclooxygenase 2; Cu^2+^, copper (II) ion; CVD, cardiovascular disease; DCF, dichlorofluorescein; DiI, 1,1-dioctadecyl-3,3,3,3-tetramethylindocarbocyanine perchlorate; DCFH-DA, 2,7-diclorofluorescein diacetate; DMSO, dimethyl sulfoxide; EC, endothelial cells; EDTA, ethylenediaminetetraacetic acid; ELISA, enzyme-linked munosorbent assay; ERK, extracellular signal-regulated kinases; FAK, focal adhesión kinase; Fu, fukugiside; GC, gas chromatography; GM-CSF, granulocyte-macrophage colony-stimulating factor; H/E, hematoxylin/eosin; HFD, high-fat diet; HPLC, high-performance liquid chromatography; IHC, immunohistochemistry; IL, interleukin; iNOS, inducible nitric oxide synthase; LDH, lactate dehydrogenase; LDL, low-density lipoprotein; LPS, lipopolysaccharide; MCP-1, monocyte chemotactic protein-1; MDA, malondialdehyde; MFI, mean of fluorescent intensity; MIP-1, macrophage inflammatory protein 1; Mo, morelloflavona; MS, mass spectrometry; NF-κB, nuclear factor-κB; NLRP3, nucleotide-binding domain and leucine-rich repeat containing protein 3; NRF-2, nuclear factor erythroid 2-related factor 2; OR, oil red; ORAC, oxygen radical absorbance capacity; oxLDL, oxidized LDL; PBS, phosphate-buffered saline; PGE_2_, prostaglandin E_2_; Rac1, ras-related C3 botulinum toxin substrate 1; REM, relative electrophoretic mobility; ROS, reactive oxygen species; SMCs, smooth muscle cells; SR, scavenger receptor; TBARS, thiobarbituric acid reactive substances; TCR, T-cell receptor; TE, trolox equivalents; TLR, Toll-like receptor; TNF, tumor necrosis factor; Vo, volkensiflavone.
