# Supplementary figures and images for: Natural Biflavonoids Modulate Macrophage–Oxidized LDL Interaction In Vitro and Promote Atheroprotection In Vivo
Source: Front Immunol. 2017 Aug 4;8:923. doi: 10.3389/fimmu.2017.00923 (PMC5543092; doi:10.3389/fimmu.2017.00923)

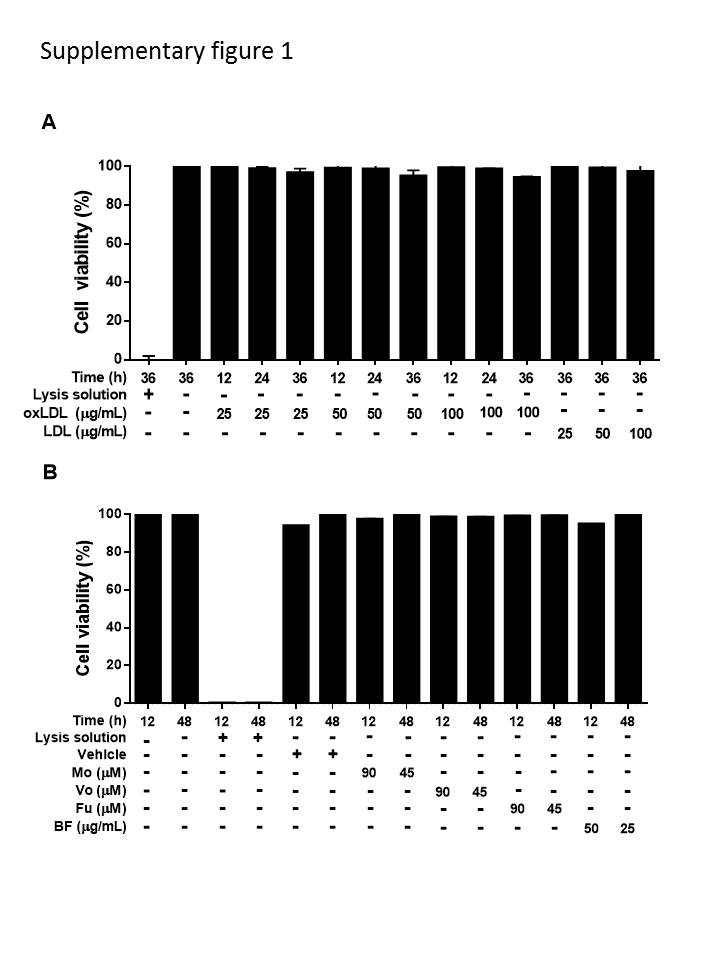

Supplement: Figure S1 — Viability of macrophage cultures in response to oxLDL or biflavonoids. Macrophages were treated during the indicated time and with the indicated amounts of oxLDL (A) or biflavonoid preparations (B) to determine cell viability by measuring LDH activity in the supernatants. Vehicle and a cell lysis solution were used as controls. Experiments were performed in triplicate, and bars represent the mean ± SD. [file image_1.tif]

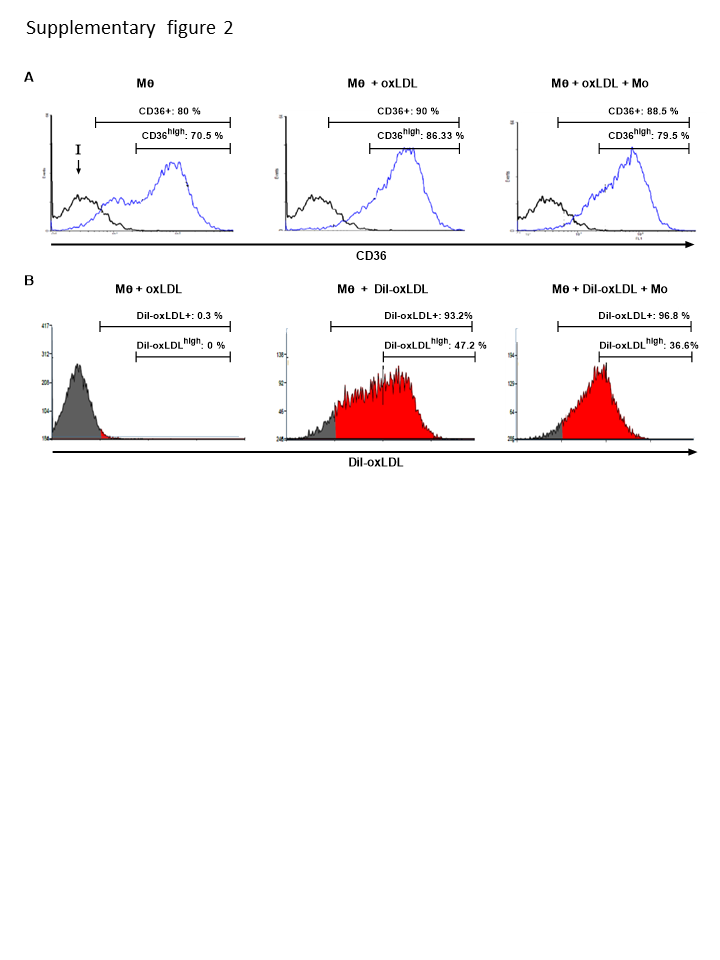

Supplement: Figure S2 — Flow cytometry analysis of CD36 surface expression and oxLDL uptake by macrophages after treatments. The effect of biflavonoids (here only Mo is shown as a representative example) on CD36 expression (A) or oxLDL uptake (B) in macrophages was investigated by flow cytometry (as explained in Materials and Methods). Representative histograms for the indicated condition are shown. Controls for CD36 expression under resting [(A), left] or oxLDL-stimulation conditions [(A), middle] are shown for comparison. Controls for non-fluorescent [(B), left] or fluorescent oxLDL (DiI-oxLDL) uptake [(B), middle] are also shown for comparison. Bars on the histograms indicate CD36+ and CD36high macrophages as well as DiI-oxLDL+ and DiI-oxLDLhigh macrophages. I: isotype controls. Mθ, macrophages. [file image_2.tif]

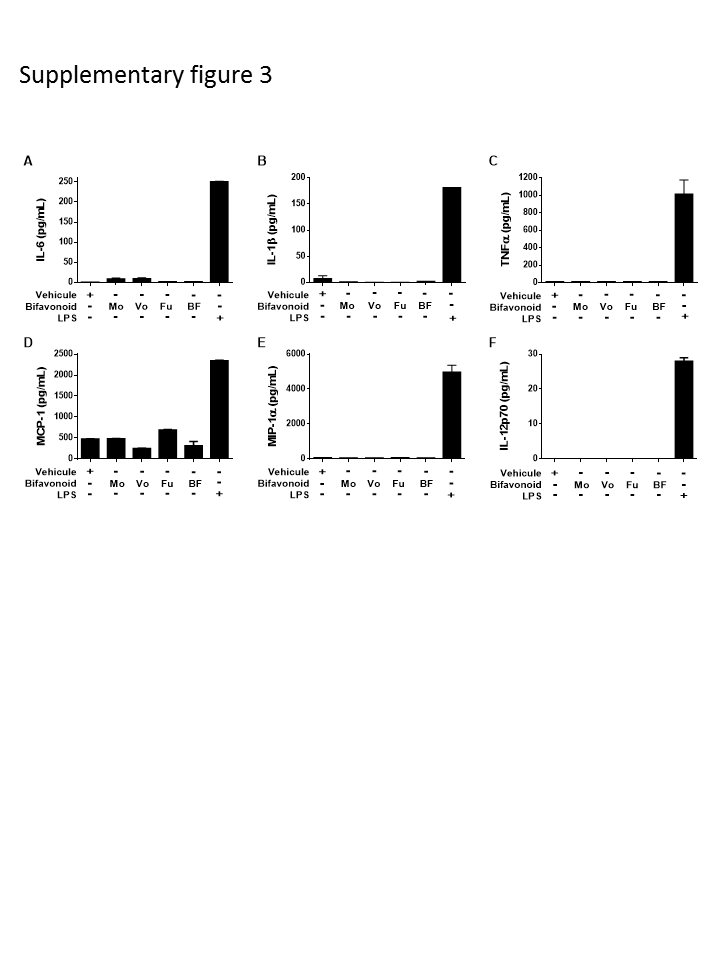

Supplement: Figure S3 — Effect of biflavonoids on proinflammatory cytokine production in resting macrophages. Macrophages were treated with biflavonoids (24 h; 45 µM for Mo, Vo, and Fu, and 25 µg/mL for BF) or vehicle and the secretion of proinflammatory cytokines IL-6 (A), IL-1β (B), TNFα (C), MCP-1 (D), MIP-1α (E), and IL-12p70 (F) was evaluated by Luminex. LPS (10 μg/mL)-treated macrophages were used as positive control. Experiment was performed in triplicate and bars represent the mean ± SD. [file image_3.tif]

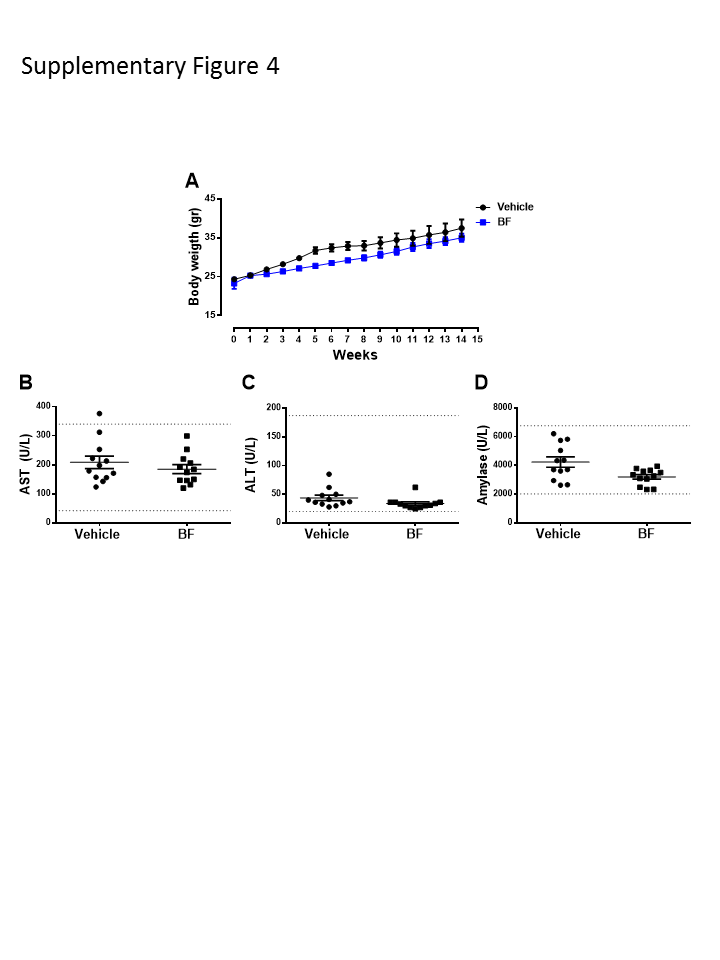

Supplement: Figure S4 — Treatment with Garcinia madruno’s BF does not alter mouse body weight or clinical biochemistry parameters. ApoE−/− mice were treated i.p. with the biflavonoid fraction (70 µg/kg), as shown in Figure 6A. Total body weight was monitored weekly and compared to vehicle-treated mice (A). Serum samples were obtained from animals at the end of treatments and the levels of aspartate transaminase (AST) (B), alanine transaminase (ALT) (C), and amylase (D) were determined. Body weight results are shown as the mean ± SEM, whereas biochemical parameters of mice are shown individually with bars indicating the mean ± SEM. No significant differences were found between the two groups. Dotted lines represent normal reference ranges for each analyte. [file image_4.tif]

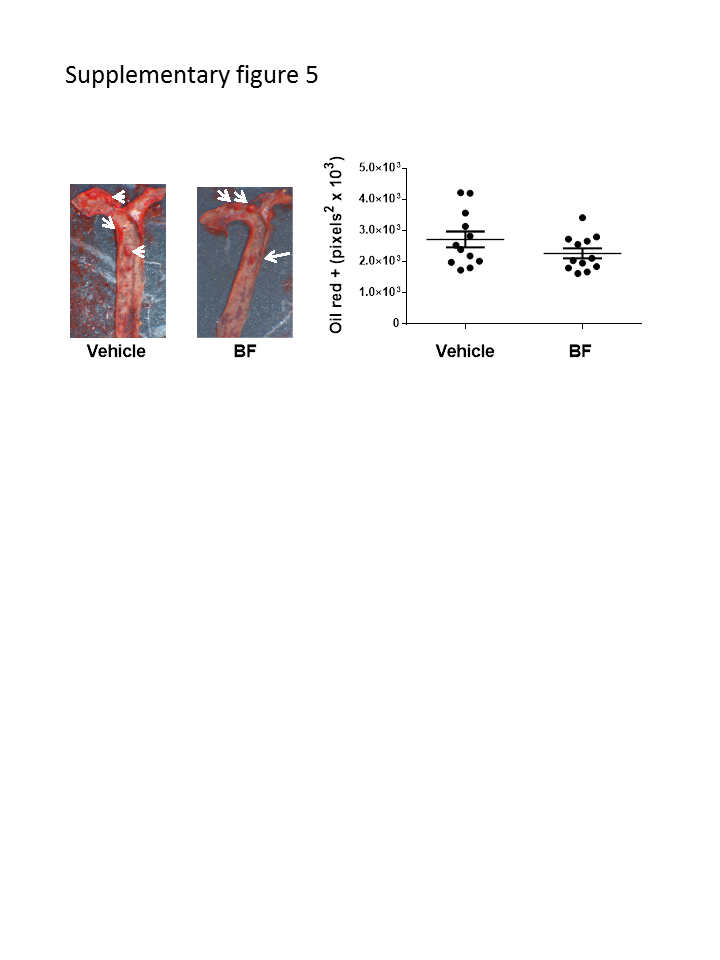

Supplement: Figure S5 — Evaluation of the effect of BF treatment on aortic lipidic deposition in ApoE−/− mice. ApoE−/− mice were treated i.p. with the biflavonoid fraction (70 µg/kg), as shown in Figure 6A. Vehicle-treated mice were used as negative controls. After sacrifice, aortas (from ascendant to abdominal) were removed, opened longitudinally, and stained with Oil Red O. Oil Red+ positive areas were calculated with the aid of image analysis software. Results are shown individually with bars indicating the mean ± SEM. No significant differences were found between the two groups. [file image_5.tif]
